# 1773. Complement level measurements for children in England suggest a possible unreported Post Streptococcal Glomerulonephritis Outbreak

**DOI:** 10.1093/ofid/ofad500.1602

**Published:** 2023-11-27

**Authors:** Rachel Dale, Ania Manson, Hoi-Ping Mok, Dinakantha Kumararatne, Effrossyni Gkrania-Klotsas

**Affiliations:** Department of Clinical Immunology Addenbrooke's Hospital, Cambridge, England, United Kingdom; Department of Clinical Immunology Addenbrooke's Hospital, Cambridge, England, United Kingdom; Department of Infectious Diseases, Cambridge University Hospitals NHS Trust, Cambridge, England, United Kingdom; Addenbrooke's Hospital, Cambridge, England, United Kingdom; Addenbrooke's Hospital, Cambridge, England, United Kingdom

## Abstract

**Background:**

Poststreptococcal glomerulonephritis (PSGN) is caused by prior infection with specific strains of group A streptococcus (GAS). PSGN is commonly characterised by significant complement C3 depression and maintained C4 levels, especially in children. The 2022-23 GAS outbreak has been associated with childhood morbidity and mortality in the UK due to invasive GAS infections but no PSGN outbreak has been described.

**Methods:**

We reviewed the laboratory requests for complement C3 and C4 measurements received by the main East of England immunology laboratory for the 48 months between April 1^st^, 2019 and March 31^st^, 2023 and for patients under the age of 18 at the time of the request. We collected demographic information as well as concurrent anti antistreptolysin and anti-DNAase B antibody levels. We excluded 4 individuals who had only marginally decreased C3 levels (less than 10% drop from lower limit of normal).

We assigned cases as possible PSGN if C3 was low, C4 was normal and antistreptolysin serologies were not obtained. We assigned cases as probable PSGN if C3 was low, C4 was normal and antistreptolysin and anti-DNAase B antibody levels were elevated.

**Results:**

24 unique patients aged 0-18 were identified (18 boys and 6 girls), 8 possible and 16 probable PSGN cases. All but two of the probable cases were boys. There were no cases of possible or probable PSGN between 9.3.20 and 20.3.22. Half of the observed cases have been observed within the last 12 months, mirroring the reported invasive GAS incidence in England.

Table of possible and probable Post Streptococcal Glomerulonephritis cases, East of England Immunology Laboratory

**Figure:**
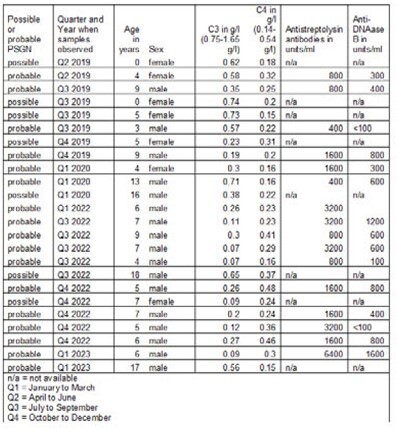

**Conclusion:**

PSGN is unusual in industrialized countries. The monitoring of C3 and C4 complement level requests suggests a possible undiagnosed concurrent to the GAS PSGN outbreak in the UK and the circulation of possible nephritogenic strains.

**Disclosures:**

**All Authors**: No reported disclosures

